# Human Occupancy Detection via Passive Cognitive Radio

**DOI:** 10.3390/s20154248

**Published:** 2020-07-30

**Authors:** Jenny Liu, Huaizheng Mu, Asad Vakil, Robert Ewing, Xiaoping Shen, Erik Blasch, Jia Li

**Affiliations:** 1 Department of Electrical and Computer Engineering, Oakland University, Rochester, MI 48309, USA; huaizhengmu@oakland.edu (H.M.); avakil@oakland.edu (A.V.); li4@oakland.edu (J.L.); 2 Air Force Research Lab, Wright Patterson Air Force Base, Dayton, OH 45433, USA; robert.ewing.2@us.af.mil; 3 Department of Mathematics, Ohio University, Athens, OH 45701, USA; shenx@ohio.edu; 4 Air Force Research Lab, Rome, NY 13441, USA; erik.blasch.1@us.af.mil

**Keywords:** human occupancy detection, passive cognitive radio, feature selection, adaptive spectrum sensing, reconfigurable software defined radio

## Abstract

Human occupancy detection (HOD) in an enclosed space, such as indoors or inside of a vehicle, via passive cognitive radio (CR) is a new and challenging research area. Part of the difficulty arises from the fact that a human subject cannot easily be detected due to spectrum variation. In this paper, we present an advanced HOD system that dynamically reconfigures a CR to collect passive radio frequency (RF) signals at different places of interest. Principal component analysis (PCA) and recursive feature elimination with logistic regression (RFE-LR) algorithms are applied to find the frequency bands sensitive to human occupancy when the baseline spectrum changes with locations. With the dynamically collected passive RF signals, four machine learning (ML) classifiers are applied to detect human occupancy, including support vector machine (SVM), k-nearest neighbors (KNN), decision tree (DT), and linear SVM with stochastic gradient descent (SGD) training. The experimental results show that the proposed system can accurately detect human subjects—not only in residential rooms—but also in commercial vehicles, demonstrating that passive CR is a viable technique for HOD. More specifically, the RFE-LR with SGD achieves the best results with a limited number of frequency bands. The proposed adaptive spectrum sensing method has not only enabled robust detection performance in various environments, but also improved the efficiency of the CR system in terms of speed and power consumption.

## 1. Introduction

The field of human detection has many important applications, ranging from autonomous vehicle safety [1], smart building surveillance [2], and site security [3], to critical disaster relief operations. Many solutions have been developed to solve the problem of human detection. The existing human occupancy sensing modalities include a visual camera [4], as well as lidar [5], radar [6,7], infrared [8], and ultrasonic sensors [9]. These modalities all have their own individual strengths and weaknesses. Cameras, for example, are capable of providing detailed feature information, which is suitable for human subject identification and tracking, but can be restricted by factors such as lighting and perspective. Optical modalities, such as cameras, can be considered invasive and may generate privacy concerns. Lidar and radar systems are expensive, and both require signal emitters. The existing wireless systems can be interfered by the actively emitted signals. The installation angle and position are very important factors that must be considered when installing human detection devices such as infrared, ultrasonic sensors, lidar, and radar. These modalities are prone to being physically obstructed or jammed. Therefore, it will be beneficial to develop a non-polluting, passive, and low-priced solution to human occupancy detection (HOD).

Lidar, radar, and ultrasonic sensors fall into the active sensing category, which includes a transmitter sending out a signal to be bounced back off the target and a receiver gathering the data upon its reflection. An example is micro-Doppler radar to discern humans from wildlife [10]. Opposite from active sensing, passive sensing techniques only detect or respond to a certain type of input from the physical environment, such as vibrations, light, radiation, heat, or other phenomena occurring in the subject’s environment. Passive sensing comes with the inherent advantage of not requiring an active signal source, and thus cannot be detected by observed parties as it only receives data. Compared to active modalities, implementing countermeasures against a passive modality becomes difficult, as rather than relying on a transmitter whose activity might be detected with equipment, passive modalities instead exploit information that can be collected without an active signal source. Several such examples of passive sensing-based technologies include photographic, thermal, electric field, chemical, infrared, and seismic signatures. For example, an innovative photographic sensor was used to accurately control the defrosting process for a commercial size air source heat pump [11]. In the research [12], wildlife was detected by thermal cameras so that they could be protected from injuring and killing by the agriculture machinery. A mechanical seismic sensor system designed from paired geophones measures the field rotation rate [13]. A passive radar system based on Wi-Fi transmissions was investigated on a two-dimensional target estimation problem [14].

Passively sensing radio frequency (RF) signals has multiple benefits, such as utilizing less of the already crowded spectrum, avoiding third-party detection, and reducing power requirement. Our cognitive radio human occupancy detection over RF analysis (CRhodora) system does not depend on any specific wireless signal types such as Wi-Fi or a cell network. Passive wireless signals are available almost anywhere except in extreme environments, such as under the sea.

The proposed research is based on the hypothesis that human subject can leave signatures on the spectrum and the spectrum alteration can be detected. The spectrum variation reflects the changes in space occupancy status; thus, a human subject can be detected by analyzing the RF data. Human occupancy in an enclosed space was successfully detected via deep learning of passive RF data in our earlier research [15]. Our initial experimental results indicated that the variation in the baseline environment spectrum caused by human occupancy can be detected by a convolutional neural network (CNN). To the best of our knowledge, it was unknown how human occupancy changes the spectrum sensed by CR before our study. To attack this problem, machine learning (ML) is utilized in our research. ML has been widely used on RF data analysis due to it intrinsic capability of learning [16,17,18,19]. ML can automatically learn the pattern by observing the labeled RF data and obtain the desired knowledge. The well-trained ML model can make good decisions to detect occupancy based on the RF samples provided, as has been examined in our initial research [15]. In order to avoid spectrum changes caused by the irrelevant electronic devices carried by human subjects, only a laptop, a software-defined radio (SDR) used to collect the data, and a personal cell phone were powered on during data collection in the enclosed space. When all the cell phone network frequency bands were excluded in training and detection, the CNN could detect human subjects accurately. Furthermore, two-month data were collected almost every day from 6 a.m. to 12 a.m. with a random occupancy status. The CNN was tested against a different time frame to detect human subjects and it performed very well. The experiment’s results showed that the CNN model detected the alteration of the spectrum caused by human subjects but not the spectrum fluctuation in a different time frame.

However, the original system can only work in a fixed location and must use the spectrum of a large number of frequency bands. To make the system more robust and efficient, an enhanced HOD system is developed and presented. In the present design, a reconfigurable software-defined radio (SDR) is used to collect RF data in three enclosed spaces, including a study room in a single-family house, a bedroom of an apartment, and a five-seat car. An unsupervised feature selection algorithm based on principal component analysis (PCA) and a supervised feature selection algorithm via recursive feature elimination with logistic regression (RFE-LR) adaptively select the sensitive frequency bands from the whole spectrum during data acquisition. Four traditional ML classifiers are trained with the power of the selected frequency band to detect human occupancy.

The frequency band in a normal environment is widely distributed from 500 KHz to 8.4 GHz. It is not economic or feasible to use full band data for HOD. Passive wireless signals cannot be controlled as the spectrum changes over time and is different from location to location. Per spectrum observation recorded with and without human occupancy, certain frequency bands are sensitive for human detection. These sensitive frequency bands should be identified in different environments and automatically determined to eliminate human effort. Cognitive radio (CR) is an adaptive intelligent radio technology that enables the radio to automatically sense the surrounding wireless spectrum and reconfigure its parameters to improve its operating behaviors. CR is the ideal candidate to accomplish dynamical frequency band selection per its reconfigurable characteristic and to proactively adapt to different environments.

Due to the constantly changing wireless environment, a feedback loop control mechanism is needed to maintain optimal detection performance. To design the control loop, an online training approach is depicted as the following. A trained ML model that can detect human occupancy in an environment is established as the base model. Online training is applied on this base model by retraining it with the newly collected and dynamically selected RF band data at a regular basis, depending on the fluctuation level and changing frequency of the wireless signals. The model is updated over time to maintain its detection accuracy.

A CNN was used in our initial research work, which provided very encouraging results [15]. However, a CNN consumes a significant amount of data, which slows down the online training speed of the system and reduces efficiency. Small amounts of data are required to train traditional classifiers due to its elaborated algorithm and predefined parameters. Several traditional ML algorithms are utilized in the CRhodora system, which achieve a very promising performance. The contributions of this research are listed as follows.

- Adaptive spectrum sensing via a reconfigurable CR is applied to HOD.
- Online training enhances the system robustness for real-time performance.
- Results demonstrate traditional classifiers achieve a better performance for human detection, using much less training samples and number of frequency bands than the CNN.

The rest of this paper is organized as follows. Section 2 presents a brief introduction on related existing HOD research work. Section 3 details the CRhodora methods, including the technical approach and the design of the experiments. Section 4 provides the experimental results and discussions. Finally, Section 5 concludes the paper and points out future research directions.

## 2. Background

### 2.1. Related Work

#### 2.1.1. Human Occupancy Detection

Different technologies have been developed for human occupancy detection, or sometimes referred to as occupancy detection, including wireless detection and video surveillance. During the mid-90 s, the subject of HOD began with infrared sensing [8]. Recently, passive wireless detection became popular as a wireless transceiver was not required to be carried by a human [16]. Li et al. used RFID tags in their experiment for human detection and behavior classification instead of passive RF [17]. Other systems depend on a Wi-Fi network to identify the common occupant activities from the Wi-Fi channel state information measurements [20]. Lv et al. made use of an active emitter to send wireless signals rather than using passive RF to quantify the quality of human actions via RF wireless signals [18]. Detecting objects for airspace surveillance by passive RF data was described in [21], but has not been applied to human detection in previous studies. Sparse vibration sensors estimated the room-level building occupancy status by extracting human footsteps from the ambient vibrations [22]. This solution proposed by Pan et al. was restricted by the sensor installation location to count the entering and leaving times from rooms. HOD inside a vehicle was addressed by Birch et al. through color image segmentation techniques [23]. Shih et al. focused on human subject detection in a building by using a camera network [24]. Both solutions are not desirable when privacy is a concern. In order to compensate for the solutions mentioned above, an occupancy detection solution is desired that should not depend on specific types of wireless signals nor introduce any concern of privacy. To make the system environment friendly and reduce the cost, the system should not emit active signals or occupy the limited communication channels. Furthermore, the deployment of the detection devices should be simple and adaptable.

#### 2.1.2. Cognitive Radio

A software-defined radio (SDR) is a radio communication system that utilizes a group of technologies, including hardware and software. Some or all functions of the radio are reconfigurable through software or firmware that are operated on the programmable processors. SDR has many applications in various fields, such as spectrum monitoring [19], RF transmitter identification [25], and other areas. For example, it was used as a receiver to estimate a mobile station’s location through received signal strength [26]. Bonoir et al. applied SDR to remote wireless tomography in their experiment [27]. In their research work, SDR was used to recognize gestures through Wi-Fi signals by Zhang et al. [28]. CR has evolved from SDR by adding additional functions, including sensing its environment, tracking changes, and reacting upon its findings by reconfiguring its setting. As described by Jondral, CR emerged in recent decades due to the rapid deployment of new wireless devices and applications [29]. The inefficient usage of limited spectrum resources by the fixed channel allocation policy urges this innovative technology to be applied quickly and widely. CR enables the development of dynamic spectrum access network that can utilize the spectrum and energy more efficiently in an opportunistic fashion and void the inference with licensed users [30]. A general metric is proposed by Wang et al. to facilitate the configurable balanced trade-off between spectral efficiency and energy efficiency for CR [31]. Liu et al. proposed a cluster-based cognitive industrial internet of things to improve the spectrum sensing and the performance of transmission through CR [32]. Power consumption can be saved by actively predicting the channel utilization status through sensing the spectrum with CR device versus continually scanning the wireless environments [33,34]. Furthermore, reinforcement learning was applied by Lin et al. to power allocation of the transmission channel and the control channel in a CR network, reducing the wasting of power [35]. Energy can be saved by incorporating the CR communication network with the smart grid, which automatically monitors and controls grid activities [36]. Joshi et al. surveyed CR wireless sensor networks and its potential application areas to military and security, healthcare, home appliances, real-time surveillance, transportation and vehicular networks, and so on [37]. The encouraging results of these existing applications indicate that CR can be an ideal candidate for HOD via passive RF sensing.

#### 2.1.3. Feature Selection

There are three common elements that classification is based on, namely, signals, features, and decisions. Processing all the signals is expensive, while decisions lack completeness, so most approaches seek feature analysis. In ML, feature selection is the process to automatically or manually determine features for decision-making. Feature selection can remove the redundant or irrelevant features in the data without losing much of the information. Feature selection can simplify the model, shorten the training time, and further enhance model generalization. The confidence (or credibility) of classification can be improved by dynamically determining how many features are necessary and which features are salient. The feature selection process falls into three categories, supervised, semi-supervised, or unsupervised, depending on the availability of the labels of the data, which are fully available, partially available, or none, respectively. Dynamic feature selection is a widely popular technique to demonstrate efficient and adaptive solutions using clustering algorithms applied on RF data. Recent books highlight the advantages of ML and deep learning to RF imagery and communications data [38]. In the real time system, radio modulations were properly classified by only selecting a small portion of spectral correlation density that can be used to classify signals without the need for system synchronization [39]. Feature selection was identified as the core step by Wang et al. to secure wireless transmission via a radio frequency distinct native attribute [40]. The indoor location estimation was optimized by adding the feature selection phase to the methodology, which was performed through the genetic algorithm (GA) [41]. All the research works mentioned above indicate that ML can benefit from the feature selection technique.

In our initial research [15], a total of 1447 frequency bands were scanned by the SDR to train a CNN for HOD. The results indicated that certain features from the very-high-dimensional data can be selected for HOD without performance sacrifice. The data stream in our experiment is high-dimensional where the feature selection can be applied to simplify the classification, accelerate the training process, and enhance model generalization. Since the passive RF data change over time and location, the frequency bands used for occupancy detection are dynamically selected to adapt the changing environments. Both supervised and unsupervised feature selection algorithms are adopted in our experiments and the comparative occupancy detection results are obtained using the frequency bands selected. The performance of the system in this paper improves previous results by keeping a very small portion of the frequency bands. 

### 2.2. Advantages

In this paper, we propose a cognitive radio human occupancy detection over RF analysis (CRhodora) system. The system measures the spectrum in an enclosed space using SDR by scanning from its lowest frequency to its highest frequency. Labels are assigned according to human occupancy status. PCA and RFE-LR-based algorithms are applied to dynamically select the frequency bands that are sensitive to HOD and reconfigure the CR. The average power of the selected frequency bands and corresponding labels are used to train the ML classifiers. Finally, the trained classifier is used for HOD in real time. There are several advantages offered by this system. Due to the absence of invasive sensors, such as cameras, privacy would not be as much of a concern. A reconfigurable CR significantly reduces the power consumption. With the absence of an active signal, the system is environmentally friendly. The low-price SDR makes it a very affordable system. The system can maintain a robust performance in different locations and times by adaptive spectrum sensing. The system shortens the time needed for system deployment as the bands are selected automatically without human interaction. Finally, it is data efficient and interpretable using classic ML models instead of deep learning neural networks.

## 3. Methodologies

**Figure 1:**
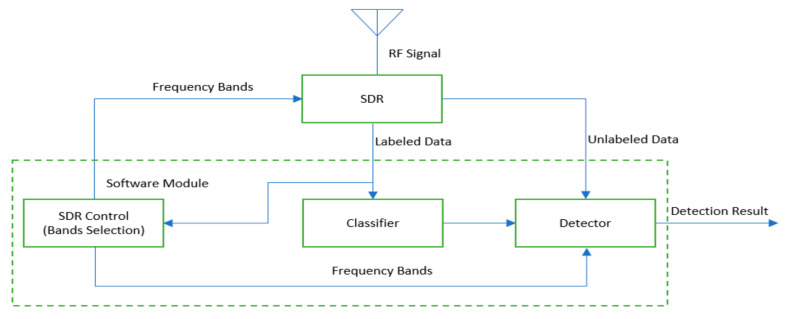
The cognitive radio human occupancy detection over RF analysis (CRhodora) system.

The proposed CRhodora system includes a receiving antenna, an SDR, and a software module that detects human subjects, and reconfigures the SDR for optimal performance. The system diagram is depicted in Figure 1. The RF signals are collected from enclosed spaces. In the initial stage, the SDR is configured by an SDR control to scan the whole spectrum in its frequency range and the collected data is labeled. The labels associate the collected RF signal with the corresponding human occupancy status. Frequency bands that are sensitive to human occupancy are selected after enough samples of the whole spectrum are collected. The SDR is reconfigured by the SDR control module to scan the selected frequency bands only. Next, the classifier is trained with the selected frequency bands samples to detect human occupancy. The detector uses the trained classifier and the passive RF signals to continuously monitor human occupancy. The frequency bands selection and classifier are updated periodically in a user-specified time interval, so that the system can adapt to the spectrum varying with time and location. Finally, the detector is updated with the adaptively trained classifier and uses the selected frequency bands for detection.

The CRhodora approach is explained further in the following subsections as RF signal acquisition, RF signal pre-processing, adaptive spectrum sensing, and classifier training.

### 3.1. RF Signal Acquisition

To eliminate the contamination of the data from irrelevant electronic devices, only the laptop and SDR used to collect the data and a personal cell were powered on in the enclosed space during data collection. The laptop and SDR always worked regardless of the occupancy status. To simulate the real-life environment wherein people carry their cell phones in most situations, and to make sure our system does not depend on the signals emitted by the cell phone, the cell phone was left power on or off in the enclosed space randomly, regardless of the occupancy status. A low-cost software-defined radio, RTL2832U, was used to collect the RF raw data at three separate locations, including a study room in a single family house, a bedroom in an apartment, and a car parked in an open space, with and without a human subject occupying the location. When the data were collected, the program automatically assigned the labels to RF raw data. During a full band scan, the spectrum was continuously scanned by the SDR with an even step size of 1.2 MHz from the lowest frequency 24 MHz to the highest frequency 1760 MHz. Passive RF signals, such as FM, TV, and cellular signals, were expected in the spectrum and vary with location and time. Passive RF data collection was described in Table 1. The data collected through a full band scan is referred to as a full band sample. One full band sample contains the raw data of 1447 frequency bands. The sampling rate is 2.4 MHz and one collection duration per frequency band is 2 ms. A total of 4800 samples per frequency band were collected at a sample rate of 2.4 MHz during each 2 ms. The sample rate of 2.4 HMz was chosen in our experiment because it is the verified highest sample rate at which the regular universal serial bus (USB) controllers do not lose samples although the theoretically possible sample rate is 3.2 MHz. Two milliseconds per frequency band were adopted so that a sufficient number of signals can be collected to maintain the detection accuracy and the system can be fast enough to monitor the occupancy status in real time.

**Figure 2:**
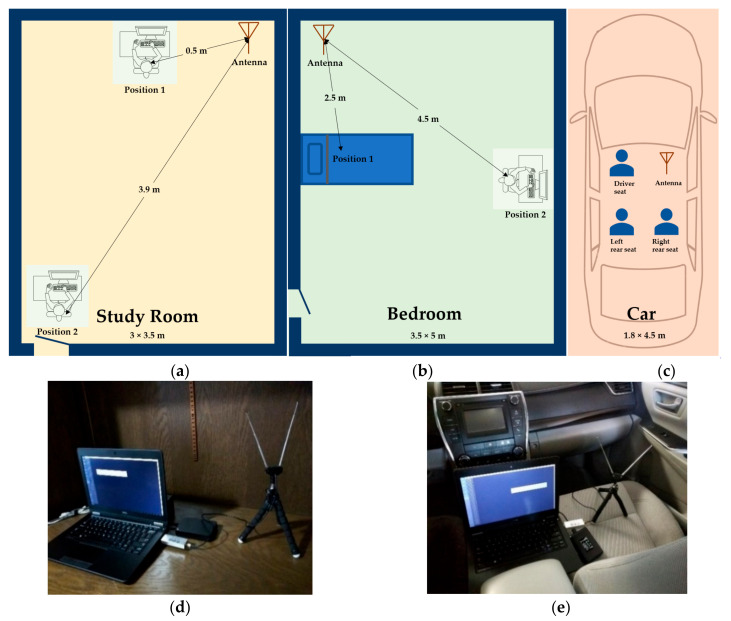
Data collection environment and hardware setup: (**a**) study room; (**b**) bedroom; and (**c**) car. (**d**) SDR and the antenna set up in the study room; and (**e**) SDR and antenna set up in the car.

At each experiment location, the antenna was placed at a fixed position with fixed directions. A human subject can occupy different positions in the enclosed space. Figure 2 illustrates the data collection environments and antenna setup. The antenna is placed at the corner of the study room and the bedroom, and at the front passenger seat in the car. A human subject stays at a position without walking and other significant motions during data collection. In the study room, the distance between Position 1 and the antenna is around 0.5 m and the distance between Position 2 and the antenna is 3.9 meters. For distances in other experiments, please refer to Figure 2. A total of 150 full band samples were collected without human subjects at each location and a total of 450 full band samples were collected at these three different locations. Then, 150 full band samples were collected when a human subject presents at a position in that enclosed space and without other human subject present at the same time at that location. The same data collection was performed for each position of each location. In total, 300 full band samples were collected in the study room, 300 full band samples were collected in the bedroom, and 450 full band samples were collected in the car with a human present. To eliminate the impact of spectrum variation among different timeframes in the day, the RF data collection with and without a human subject occupying the space was performed at a similar time period of the day at each location. For example, the data collection in the car was only conducted in the afternoon time from 1 p.m. to 6 p.m. It takes a few days to collect the data for each location. Two identical SDRs were used to collect the data to reduce the data collection time and eliminate the device dependency.

In order to verify how well the system works at different locations and different environments, experiments were carried out at several locations. They are Position 1 in the study room (StRmP1), Position 2 in the study room (StRmP2), Position 1 in the bedroom (BdRmP1), Position 2 in the bedroom (BdRmP2), driver seat in the car (CrP1), left rear seat in the car (CrP2), and right rear seat in the car (CrP3). The system detects human occupancy but does not estimate the subject’s location nor the exact number of human subjects.

### 3.2. RF Signal Pre-Processing

To estimate the power spectrum, the average power per frequency band was calculated. The number of samples per frequency band, denoted by  N, was 2400. $p\left(f\right)$ is the average power of the frequency band centered at f and is calculated as below.

(1) $$p\left(f\right)=10\;*\;\frac{{log}_{10}\left(\sum_{i=1}^{N}a_{i}{\left(f\right)}^{2}\right)}{\frac{N}{2}}$$

where $a_{i}\left(f\right)$ is the amplitude of the i-th intermediate frequency signal received by the SDR at the frequency band of f. Let M be the number of full band samples, which is 150 in our experiment. $p_{avg}\left(f\right)$ is the average power spectrum estimated over M full band samples calculated by $p_{avg}\left(f\right)=\sum_{j=1}^{M}p_{j}\left(f\right)/M$, where j is the index of the power spectrum samples. 

**Figure 3:**
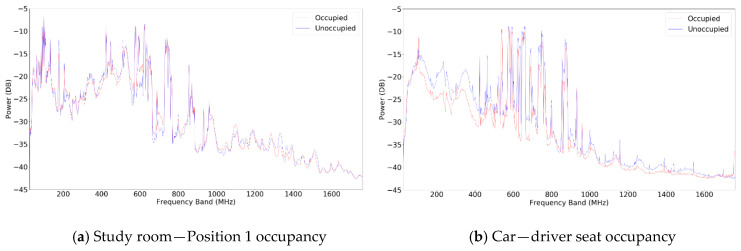
Average power spectrum of occupied vs. unoccupied. (**a**) Study room—Position 1 occupancy. (**b**) Car—driver seat occupancy.

Snapshots of the power spectrum at different locations are shown in Figure 3. The red line is for the occupied situation, while the blue line is for the unoccupied situation. There are noticeable differences between the spectrums of the occupied and unoccupied scenarios at each location. The degree of variation between the two scenarios is location dependent. For example, the spectrum variation is larger inside the car than that of the study room. The results are probably affected by factors such as the body mass of the human subject, the materials inside of the enclosed space, the spectrum, or other unknown factors. For example, the metal material in the car may cause the large variation. The cause and the environmental variation shall be further investigated in future research.

### 3.3. Adaptive Spectrum Sensing

The power spectrum measured by the SDR varies with time and location. The devices that transmit signals can be added or removed and it is difficult to predict the precise transmission usages. For example, more wireless channels are used during daytime when there are more human activities, while less signals are transmitted during the night. Many radio stations only transmit at certain hours every day. The spectrum also varies by location as the RF signals tend to be sparser in rural areas than in crowded cities. The Wi-Fi is stronger in places where more people tend to visit more frequently. Even in the same location, the environment setup, such as building materials, furniture in a room, the electronic devices used, and so on, can add further variation to the spectrum. The spectrum sensing must be adaptive to these changes to guarantee robust performance. On the other hand, it is inefficient to use the whole power spectrum for occupancy detection. The prolonged scanning time per cycle leads to lower time resolution and wasted power. For these two reasons, adaptive spectrum sensing is desired to improve the robustness and efficiency of the system.

Opportunistic spectrum access through a reconfigurable CR has been well studied by many researchers [42,43,44], to adapt the constantly changing wireless environment in a real time manner, improve system performance, and reduce the power consumption. In our study, adaptive sensing is realized by dynamically selecting the frequency bands that are sensitive to HOD at various locations and time. The baseline power spectrum is adjusted accordingly.

It is well known that good feature selection can help improve classification performance [38,45,46]. The frequency band selection process aims to remove the bands that are not sensitive to human occupancy and only keep those sensitive ones. The average power of each frequency band $p_{avg}\left(f\right)$ is calculated during data pre-processing. Our observation of the measured power spectrum finds that the power of many frequency bands does not have a noticeable change between the occupied and unoccupied scenarios. This suggests that the optimal frequency band selection can result in a significant dimension reduction of data. An automatic process is desired for dynamic frequency band selection. Supervised feature selection requires labeled data while unsupervised feature selection can work with unlabeled data. For evaluation purposes, a PCA-based, unsupervised selection algorithm as well as an RFE-LR supervised selection algorithm were implemented to compare their frequency band selection results.

#### 3.3.1. PCA-Based Frequency Band Selection

The classic principal component analysis (PCA) is an algorithm that can reduce dimensionality of a dataset and increase the interpretability of data while minimizing information loss. It has been widely applied in data analysis, data processing, and dimensionality reduction. However, classical PCA methods are not associated with a probability density and cannot be extended to a mixture of probabilistic models, which is usually the case for unsupervised learning and feature selection. To overcome this limit, a number of approaches have been attempted to formulate the mixture models. Most of these approaches are two-stage procedures with the first step partitioning the data space followed by estimation of the principal subspace within each partition, i.e., local PCA. Tipping and Bishop proposed a probabilistic PCA (PPCA) model, which can be naturally extended to a mixture of local PCA models [47]. The PPCA method estimates the probabilistic model by the maximization of a pseudo-likelihood function and avoids an explicit two-stage algorithm. In this paper, we apply the PPCA algorithm with $p\left(f\right)$ as the input features to extract the principal components from the power spectrums of the different locations.

As each principal component is a linear combination of all the original frequency bands, if the system directly uses the extracted principal components as features, the interpretation of the results and subsequent spectrum sensing still has to involve all of the bands, even if only a few components are kept. So, we selected frequency bands according to their loadings in the extracted components [48]. Once the principal components were extracted, they were ranked from high to low by importance according to the variance they can explain, and the first three components were kept. Finally, k ($k\in \left[10,\;150\right]$) frequency bands with the highest absolute coefficients in the first three components were selected.

#### 3.3.2. RFE-LR-Based Frequency Band Selection

Recursive feature elimination (RFE) recursively removes the weakest feature and considers smaller and smaller sets of features until the specified number of features is reached by fitting an estimator that assigns weights to features. RFE is computationally less complex using the feature weight coefficients or feature importance when compared to sequential backward selection (SBS), which eliminates features based on a user-defined classifier or regression performance metric. RFE was applied to select features used to measure the transient stability in the power system [49]. Features which were the most significant were chosen by SBS to analyze the auditory evoked potential parameters in the presence of radiofrequency fields [50]. RFE is applied in our study to reduce the computation cost in the real-time system. Logistic regression (LR) with L2 regularization and the variation of limited-memory Broyden–Fletcher–Goldfarb–Shanno (L-BFGS) optimization [51] were chosen as the estimator when applying RFE in our research. Initially, the values of $p\left(f\right)$ of these 1447 frequency bands and corresponding 1477 labels, whose values are 1 or 0, were fed to the LR estimator. The coefficients were obtained by training the LR estimator. A certain number of frequency bands with the smallest coefficients were removed and the rest were kept. Then the first round of least significant frequency bands elimination finishes. The $p\left(f\right)$ of the remaining frequency bands and corresponding labels were used in the next round of feature elimination. The same process was repeated till the k ($k\in \left[10,\;150\right]$) frequency bands were kept. The ranking numbers were assigned during the recursive elimination process and the frequency bands were ranked from high to low by importance.

### 3.4. Classifier Training

Four traditional supervised classifiers were trained with the data of the selected frequency bands, including support vector machine (SVM), k-nearest neighbors (KNN), decision tree (DT), and linear SVM with stochastic gradient descent (SGD) training. A total of 300 full band samples collected from each experimental scenario with and without human occupancy were randomly divided into w training data set and testing data set. The training data was fed to each individual classifier and used to train the model accordingly. The input of each classifier is the list of average power of selected frequency bands and the list of the associated labels. Then these four models were trained individually for each scenario based on each band selection result, which are listed in Table 2. For example, for scenario StRmP1, 10 full band samples were randomly selected out of 150 full band samples of the occupied group, and 10 full band samples were randomly selected out of 150 full band samples of the unoccupied group. The 10 most sensitive frequency bands were selected using these 20 full band samples. The average power of these selected 10 frequency bands of 90 occupied and 90 unoccupied samples was used to train all the classifiers. The same process was repeated for a different number of full band samples and different number of selected bands, as indicated in Table 2, to find the optimal setup. For each scenario, a total of 90 experimental runs were conducted for a classifier. The different percentage of training samples over the total samples was also surveyed to identify the most efficient training strategy.

## 4. Experimental Results

In order to quantify the overall accuracy of the occupancy detection result, the actual performance was evaluated by a confusion matrix with the equations and the calculation process as follows. The evaluation parameters include the accuracy, precision, recall, and F1 score from the true positive (TP), true negative (TN), false positive (FP), and false negative (FN) results. The F1 score is used in this section to quantize the system performance unless otherwise specified.

(2) $$\mathrm{accuracy}=\frac{\mathrm{TF}+\mathrm{TN}}{\mathrm{TP}+\mathrm{FN}+\mathrm{TN}+\mathrm{FP}}$$

(3) $$\mathrm{precision}\;=\frac{\mathrm{TP}}{\mathrm{TP}+\mathrm{FP}}$$

(4) $$\mathrm{recall}\;=\frac{\mathrm{TP}}{\mathrm{TP}+\mathrm{FN}}$$

(5) $$F1=\frac{2\times \mathrm{precision}\times \mathrm{recall}}{\mathrm{precision}+\mathrm{recall}}$$

### 4.1. Frequency Bands Selected

**Figure 4:**
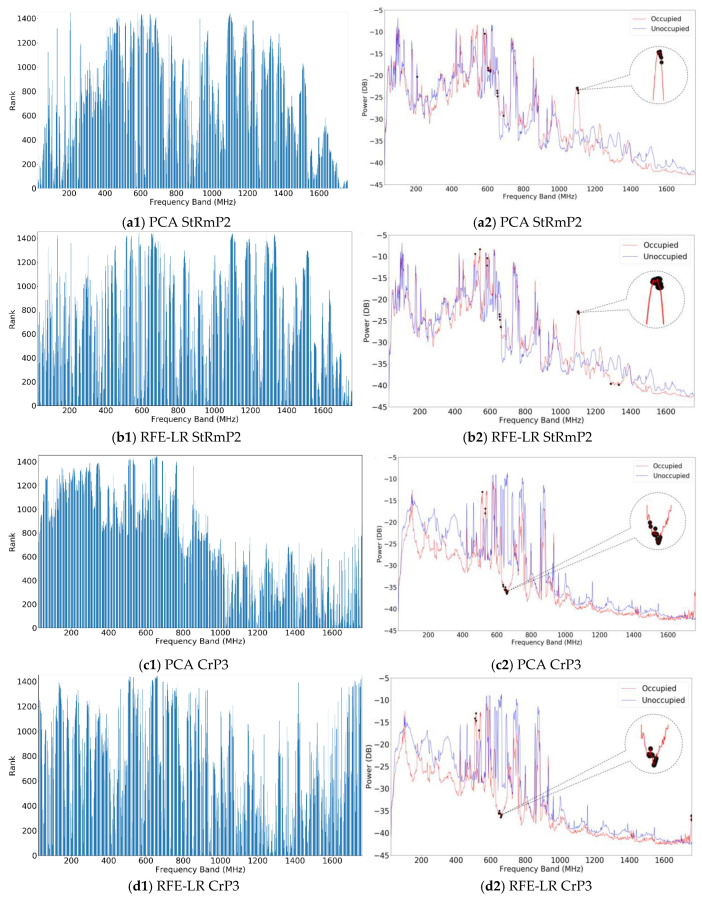
Examples of the dynamic band ranking and selection results from the PCA and RFE-LR.

To find the optimal setup of the system, different numbers of full band samples and different numbers of selected frequency bands were tested. For the number of full band samples, from 10 to 150 samples with a step of 10 samples were tested. When each number of the full band samples was tested, the frequency bands from 10 to 60 bands with a step of 10 bands were selected and used for human detection. The same process was applied in all seven scenarios. PCA and RFE-LR were used for band selection individually and the corresponding selected features were used to train the classifiers and detect occupancy. Figure 4 displays the results of the band selection of two different scenarios by the two different feature selection algorithms.

The two scenarios are StRmP2 and CrP3. The subfigures in the left column display the rank of each frequency calculated by the PCA and RFE-LR-based band selection algorithms, while the subfigures in the right column display the power spectrum marked with 30 selected frequency bands. The figures from Figure 4(a1) to Figure 4(b2) are for scenario StRmP2 and figures from Figure 4(c1) to Figure 4(d2) are for scenario CrP3. For example, Figure 4(a1,b1) depict the rank of frequency bands evaluated by the PCA and RFE-LR for the same scenario StRmP2 using 60 full band samples. The results in Figure 4 show that the PCA and RFE-LR-based algorithms produce similar ranking results. Figure 4(a2,b2) are the band selection results of scenario StRmP2. The dark dots in these two figures represent the frequency bands selected. For better visualization, the zoomed in version of certain frequencies are displayed to compare the results of the two band selection algorithms. The results show that sensitive frequency bands can be picked by both unsupervised and supervised algorithms. The frequency bands selected by the two algorithms are slightly different but have very similar clusters around 600 MHz and 1100 MHz. The ranking results and band selection results depend on the location and the spectrum variance caused by the human body. Both band selection algorithms select the frequency bands where significiant variration exists between the occupied and unoccupied spectrum. The results demonstrate that the developed adaptive sensing techniques can work as long as human subjects have RF signatures in the SDR’s frequency range. 

The cluster effect in the selected frequency bands can be detected in Figure 4 in different scenarios. Examples of selected frequency bands across all seven scenarios by PCA and RFE-LR are listed in Table 3. In these two examples, 10 frequency bands were picked by each algorithm from 40 randomly selected full band samples in the corresponding scenario—with and without human occupancy, 20 in each class. The results show that there is at least one enclosed cluster in each location. For example, in Scenarios StRmP1 and StRmP2, where data were collected in the study room, there are a few bands selected around 600 MHz. The same can be observed in the bedroom and car locations. The cluster effect is shown in the results of both band selection methods. In another example, Scenario CrP1, the frequency bands selected are between 514.8 MHz and 638.4 MHz in both Table 3a,b.

Multiple frequency bands around 1100 MHz were picked by the PCA and RFE-LR in Scenario StRmP2. Similar patterns are shown in other scenarios. The cluster effect could be related to the surrounding environment and antenna’s direction and setup. The cluster effect can be used to establish a baseline of dynamic band selection because the selected frequency bands across all the three locations have common frequencies from 500 MHz to 700 MHz. Thus, less power will be required, and the band selection time can be shortened. This cluster effect may also be useful for the study of human RF signature prediction. Electromagnetic and biological experiments can be designed to further investigate the cluster phenomenon.

**Figure 5:**
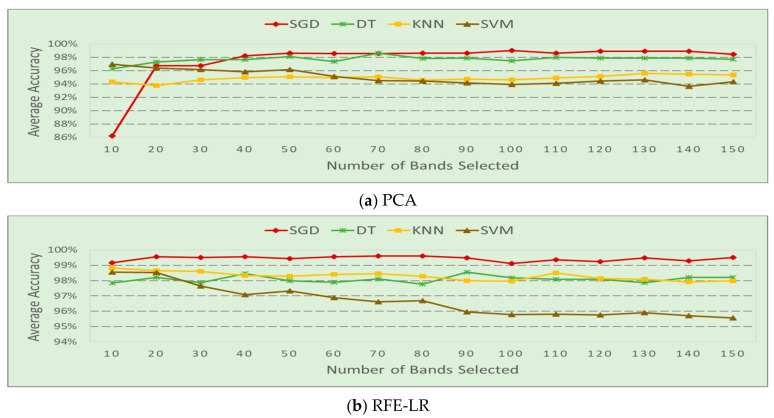
Average accuracy vs. the number of frequency bands used.

The power of dynamically selected frequency bands data was used for HOD. In order to improve the system efficiency, the number of frequency bands needed for detection was evaluated. The average occupancy detection accuracy of each classifier by using the frequency band selected by each band selection method is depicted in Figure 5. In the figure, average accuracy is calculated by the corresponding F1 score recorded during each experimental run. Let M be the number of steps of the full band samples and d be the F1 score of each experimental run; then the average accuracy of each scenario is calculated by $ds_{avg}=\left(\sum_{i=1}^{M}d_{i}\right)/M$. The average accuracy of each classifier of each band selection algorithm is calculated by $dc_{avg}=\left(\sum_{i=1}^{L}ds_{avg}\right)/L$, where L is the number of scenarios. The experiment results displayed in Figure 5 indicate that optimal feature selection policy could improve the system efficiency. The detection accuracy increases with the number of selected bands initially, and then is maintained at the same level or drops slightly after a certain number of bands selection. For example, by using the band selection algorithm PCA, the classification accuracy of model SGD increases from 86% to 98% when the number of frequency bands increases from 10 to 40. There is very limited improvement when more frequency bands are used. So, 40 can be regarded as a cutoff number in band selection by SGD. DT shows a similar trend but performs slightly worse after 70 frequency bands. The SVM works the best using only 10 bands and the performance drops continually afterwards. KNN shows improvements from 10 to 40 bands and slowly deteriorates after that. Similar trends are shown in the results of RFE-LR, but the cutoff number can be different. SGD reaches the best performance at 20 bands. DT learning does not have significant improvement after 40 bands. The performance of KNN and SVM continually drops after 10 bands. When only 10 frequency bands are scanned by the SDR, nearly 97.2% energy and time can be saved compared to using the 1447 full bands data.

**Figure 6:**
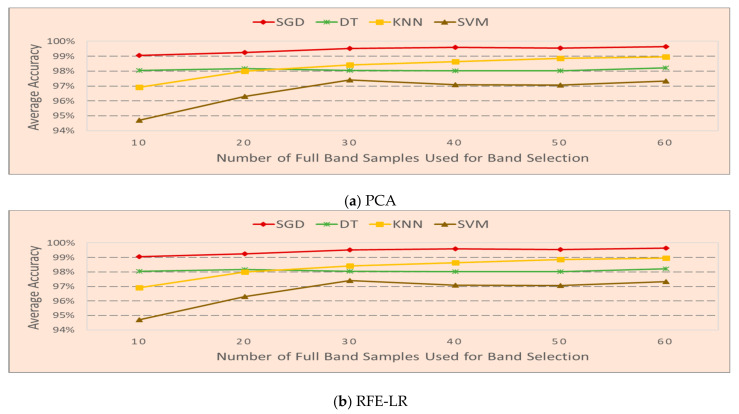
Average accuracy vs. number of samples used for bands selection.

We have also investigated how the number of full band samples affects band selection and the classifiers’ accuracy. The results are shown in Figure 6. The F1 score is used to calculate the average accuracy with a similar process above. Let *N* be the number of bands selected, and d is the F1 score obtained in each experiment. The average accuracy of each scenario is calculated by $ds_{avg}=\left(\sum_{i=1}^{N}d_{i}\right)/N$. The average accuracy of each classifier of each band selection algorithm is calculated by $dc_{avg}=\left(\sum_{i=1}^{L}ds_{avg}\right)/L$, where L is the number of scenarios. In Figure 6a, the overall trend shows that the performance increases when the number of frequency band samples used for band selection increases from 10 to 20 bands and the accuracy of all four classifiers saturates after the cutoff number of 20 by PCA-based band selection. However, in Figure 6b, which is through the RFE-LR-based band selection method, classifiers SGD and SVM reach the best performance at 30 samples and KNN shows continuous improvement till 60 samples. DT is not very sensitive to the number of samples for band selection. The overall trend in Figure 6 indicates that a very large number of full band samples used for band selection does not help in most situations and building an online training system is feasible with as little as 20 to 30 full band samples.

**Figure 7:**
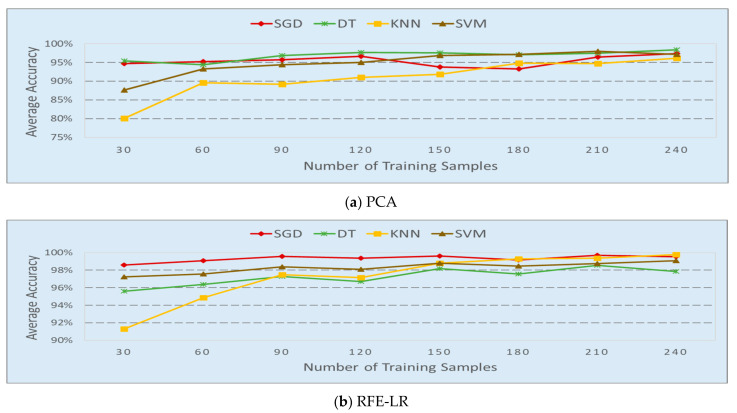
Average accuracy vs. number of samples used for classifier training.

The number of samples to train the classifiers is studied and the results are shown in Figure 7. In this study, the 60 full band samples, including 30 in the occupied group and 30 in unoccupied group, were used for band selection. Twenty frequency bands were selected by the PCA and REF-LR-based algorithms from the same frequency data samples in each scenario. The number of samples used to train the classifiers varies from 30 to 240. The F1 score was used to calculate the average accuracy. Let L be the number of scenarios and d be the F1 score of each experiment. The average accuracy of each classifier is calculated by $ds_{avg}=\left(\sum_{i=1}^{L}d_{i}\right)/L$. Each classifier shows a similar trend where the classifier’s performance improves with the increase in training samples, except DT with a PCA-based band selection method. In that case, the number of training samples does not have a significant impact to the classifier’s performance. For classifiers SGD, DT, and SVM, these are not significant improvements of accuracy, even getting a little worse after a cutoff number of 90. KNN requires 180 training samples to achieve the best performance.

### 4.2. Performance in Different Locations

We compare the classifier’s performance in different locations in this subsection. Table 4 lists the precision, recall, F1 score, and accuracy of SGD in different locations. In this example, 20 frequency bands were selected by the PCA or RFE-LR from 60 full band samples, 30 in each occupancy status, in each perspective scenario. Classifier SDG is trained to detect human occupancy. The RFE-LR-based band selection achieves better overall system performance. The detection results from the other three classifiers also indicate that the RFE-LR based band selection can lead to better detection performance.

An example of all the classifiers’ performance at different locations is presented in Table 5. In this example, 30 frequency bands were selected by the PCA or RFE-LR-based algorithms from 80 full band samples, with 40 in each occupancy status, in each perspective scenario. A total of 60% of the collected samples were used in the training and the rest used for testing. Other experiments with a different number of frequency bands selected and different number of full band samples used for band selection yield similar results.

### 4.3. Performance of Different Band Selection Algorithms

**Figure 8:**
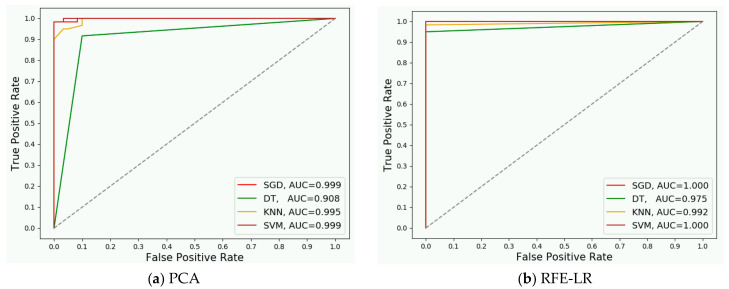
The ROC curve of human detection in Scenario StRmP1.

We evaluated how the band selection algorithm affects the classifiers’ accuracy. The detection rate and false alarm rate were measured during the experiment. The receiver operating characteristic (ROC) curves of all four classifiers are displayed in Figure 8. And correspond to PCA and RFE, separately, to select 40 frequency bands from 40 full band samples in Scenario StRmP1. The area under the curve (AUC) in these two figures indicated that the classifiers perform better using the REF selected frequency bands, except for KNN that shows a slightly lower performance.

**Figure 9:**
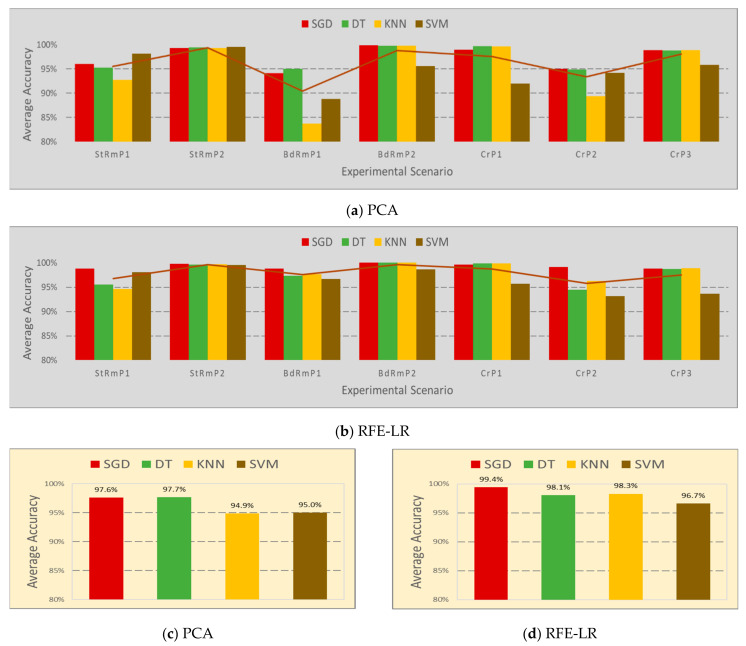
The average accuracy of human detection in difference locations.

The F1 score is used to calculate the average accuracy, which is shown in Figure 9. Let N be the number of experiments executed for each scenario, whose value is 90, and  d is the F1 score obtained in each experimental run. The average accuracy of each scenario of each band selection algorithm in Figure 9a,b is calculated by $ds_{avg}=\left(\sum_{i=1}^{N}d_{i}\right)/N$. The average accuracy of each classifier of each band selection algorithm in Figure 9c,d is calculated by $dc_{avg}=\left(\sum_{i=1}^{L}ds_{avg}\right)/L,$ where L is the number of scenarios. The average detection accuracy in each scenario in Figure 9a,b shows similar results, as studied in Section 4.2. Using the frequency bands selected by RFE-LR, the detection accuracy of each classifier achieves better result in most scenarios. More clear results are directed in Figure 9c,d. With the help of RFE-LR, the average accuracy of the KNN is improved by 3.4% from 94.9% to 98.3%, and rest of the three classifiers also show increments. 

The RFE-LR band selection algorithm helps all four classifiers to achieve better results, which is also higher than the accuracy of 95% obtained by the CNN in our initial research [15]. The highest performance is obtained by the SGD using the frequency bands picked by RFE-LR. The system performance can be improved by smartly choosing the dynamic band selection algorithm, the classifier, and other parameters, such as the number of bands selected, number of full band samples used for band selection, and the number of samples used to train the model. Figure 9 summarizes all the studies presented in the paper for a passive RF HOD.

### 4.4. Storage and Processing Evaluation

The system’s storage and processing needs were also evaluated, as the final goal of this research work is to implement all the functions on an embedded system. In the case of selecting 30 frequency bands from 60 full band samples, and then processing the data on a single core of a central processing unit (CPU)—an “AMD Ryzen Threadripper 2950X 16-Core Processor”—70.5 M bytes of memory are used, and 2.74% of the processor is utilized by the PAC. Using RFE consumes 65.2 M bytes of memory and the processor utilization rate is 2.89%, with the same case and on the same processor. So, we believe the system’s storage and processing needs can be fulfilled by a well-designed embedded system.

## 5. Conclusions

This paper proposed a new efficient, low-cost, and environmentally friendly solution to detect human occupancy in enclosed spaces via passive CR. The solution is based on a reconfigurable software-defined radio system and adaptive spectrum sensing technology. The experimental results show that the CRhodora system is capable of accurately detecting human occupancy—not only in residential rooms—but also in commercial vehicles under different settings, such as various distances between the human subject and the antenna. Frequency bands sensitive to human occupancy can be determined by both unsupervised and supervised dynamic feature selection algorithms. The supervised RFE-LR-based algorithm shows an improvement in performance over the unsupervised PCA-based algorithm. The accuracy of the occupancy status estimated by the traditional classifiers trained on selected significant features surpass the CNN on this task through the help of adaptive spectrum sensing technology. By dynamically configuring the CR and adaptively sensing the spectrum at the location of interest, the overall speed and power consumption is improved by 97.2%.

Our investigation reveals some interesting phenomenon, such as the clustering of frequency bands sensitive to the human body around 600 MHz, which requires a more thorough study. We are particularly interested in synthesizing human RF signatures for a given baseline spectrum. Such a capability to predict human RF signatures can be very useful in both security and smart building applications. The generative adversarial network (GAN) is widely used for synthesizing related signatures and will be explored in a future study. 

## Figures and Tables

**Table 1:** Software-defined radio (SDR) configuration for passive RF data collection.

Items	Description
SDR	RTL2832U
Locations	a study room, a bedroom, and a car
Data Labels	0: Unoccupied 1: Occupied
Frequency Range	24 MHz–1760 MHz
Scanning Step	1.2 MHz
Bandwidth	1.2 MHz
Sampling Rate	2.4 MHz
Duration	2 ms per frequency band
Number of Frequency Bands	1447

**Table 2:** Training setup for all scenarios and classifiers.

Scenario	Number of Full Band Samples	Number of Bands Selected	Classifier
StRmP1, … CrP3	(10, 20, … 60)	(10, 20, … 150)	SGD, SVM, KNN, DT

**Table 3:** The 10 frequency bands selected from 40 full band samples for 7 scenarios in the order from most significant to least significant.

**(a) PCA**						
**StRmP1** **(MHz)**	**StRmP2** **(MHz)**	**BdRmP1** **(MHz)**	**BdRmP2** **(MHz)**	**CrP1** **(MHz)**	**CrP2** **(MHz)**	**CrP3** **(MHz)**
180.0	206.4	1755.6	1755.6	637.2	517.2	531.6
930.0	1101.6	1758.0	1756.8	636.0	513.6	532.8
178.8	583.2	1756.8	1758.0	514.8	625.2	542.4
614.4	1102.8	1759.2	1759.2	537.6	626.4	646.8
603.6	1104.0	1754.4	621.6	516.0	624.0	645.6
612.0	1105.2	583.2	626.4	634.8	742.8	648.0
604.8	1100.4	582.0	625.2	538.8	741.6	534.0
602.4	1099.2	584.4	1754.4	638.4	740.4	537.6
177.6	654.0	580.8	622.8	584.4	692.4	649.2
176.4	614.4	452.4	624.0	633.6	693.6	636.0
**(b) RFE-LR**						
**StRmP1** **(MHz)**	**StRmP2** **(MHz)**	**BdRmP1** **(MHz)**	**BdRmP2** **(MHz)**	**CrP1** **(MHz)**	**CrP2** **(MHz)**	**CrP3** **(MHz)**
102.0	132.0	103.2	516.0	540.0	463.2	531.6
206.4	583.2	109.2	517.2	541.2	464.4	532.8
216.0	654.0	486.0	552.0	542.4	583.2	645.6
396.0	660.0	488.4	553.2	580.8	597.6	649.2
505.2	1098.0	544.8	554.4	582.0	618.0	658.8
513.6	1099.2	595.2	649.2	583.2	764.4	660.0
649.2	1100.4	624.0	650.4	634.8	768.0	661.2
650.4	1101.6	633.6	655.2	636.0	770.4	662.4
1335.6	1285.2	798.0	660.0	637.2	798.0	1755.6
1336.8	1286.4	858.0	661.2	638.4	960.0	1756.8

**Table 4:** The performance of the SGD model.

**(a) PCA**				
**Scenario**	**Precision**	**Recall**	**F1**	**Accuracy**
StRmP1	98.33%	98.33%	98.33%	98.33%
StRmP2	100.00%	100.00%	100.00%	100.00%
BdRmP1	91.67%	91.67%	91.67%	91.67%
BdRmP2	100.00%	100.00%	100.00%	100.00%
CrP1	100.00%	100.00%	100.00%	100.00%
CrP2	96.61%	95.00%	95.80%	95.83%
CrP3	100.00%	100.00%	100.00%	100.00%
**(b) RFE-LR**				
**Scenario**	**Precision**	**Recall**	**F1**	**Accuracy**
StRmP1	100.00%	100.00%	100.00%	100.00%
StRmP2	100.00%	96.67%	98.31%	98.33%
BdRmP1	100.00%	96.67%	98.31%	98.33%
BdRmP2	100.00%	100.00%	100.00%	100.00%
CrP1	100.00%	100.00%	100.00%	100.00%
CrP2	100.00%	98.33%	99.16%	99.17%
CrP3	100.00%	100.00%	100.00%	100.00%

**Table 5:** The classifiers’ performance at different locations.

**(a) PCA**				
**Scenario**	**SGD**	**DT**	**KNN**	**SVM**
StRmP1	90.48%	95.65%	90.09%	100.00%
StRmP2	100.00%	100.00%	99.16%	100.00%
BdRmP1	93.75%	96.67%	87.80%	92.31%
BdRmP2	100.00%	100.00%	100.00%	100.00%
CrP1	100.00%	100.00%	100.00%	94.49%
CrP2	96.67%	98.31%	92.86%	95.24%
CrP3	100.00%	100.00%	100.00%	97.56%
**(b) RFE-LR**				
**Scenario**	**SGD**	**DT**	**KNN**	**SVM**
StRmP1	99.17%	92.56%	91.89%	98.36%
StRmP2	100.00%	99.16%	100.00%	100.00%
BdRmP1	100.00%	97.52%	100.00%	100.00%
BdRmP2	100.00%	100.00%	100.00%	100.00%
CrP1	98.31%	100.00%	100.00%	96.77%
CrP2	100.00%	91.89%	97.44%	96.00%
CrP3	100.00%	100.00%	100.00%	96.77%
